# Multiplex genomic structure variation mediated by TALEN and ssODN

**DOI:** 10.1186/1471-2164-15-41

**Published:** 2014-01-18

**Authors:** Sanyuan Ma, Xiaogang Wang, Yuanyuan Liu, Jie Gao, Shengling Zhang, Run Shi, Jiasong Chang, Ping Zhao, Qingyou Xia

**Affiliations:** State Key Laboratory of Silkworm Genome Biology, Southwest University, Chongqing, China; State Key Laboratory of Silkworm Genome Biology, Southwest University, Beibei, Chongqing 400716 China

**Keywords:** TAL Effectors, Genomic structure variation, Chromosomal deletion, Chromosomal duplication, Chromosomal inversion, ssODN

## Abstract

**Background:**

Genomic structure variation (GSV) is widely distributed in various organisms and is an important contributor to human diversity and disease susceptibility. Efficient approaches to induce targeted genomic structure variation are crucial for both analytic and therapeutic studies of GSV. Here, we presented an efficient strategy to induce targeted GSV including chromosomal deletions, duplications and inversions in a precise manner.

**Results:**

Utilizing Transcription Activator-Like Effector Nucleases (TALEN) designed to target two distinct sites, we demonstrated targeted deletions, duplications and inversions of an 8.9 Mb chromosomal segment, which is about one third of the entire chromosome. We developed a novel method by combining TALEN-induced GSV and single stranded oligodeoxynucleotide (ssODN) mediated gene modifications to reduce unwanted mutations occurring during the targeted GSV using TALEN or Zinc finger nuclease (ZFN). Furthermore, we showed that co-introduction of TALEN and ssODN generated unwanted complex structure variation other than the expected chromosomal deletion.

**Conclusions:**

We demonstrated the ability of TALEN to induce targeted GSV and provided an efficient strategy to perform GSV precisely. Furthermore, it is the first time to show that co-introduction of TALEN and ssODN generated unwanted complex structure variation. It is plausible to believe that the strategies developed in this study can be applied to other organisms, and will help understand the biological roles of GSV and therapeutic applications of TALEN and ssODN.

**Electronic supplementary material:**

The online version of this article (doi:10.1186/1471-2164-15-41) contains supplementary material, which is available to authorized users.

## Background

Genomic structure variations (GSVs), including chromosomal deletions, duplications, inversions, insertions and translocations, are one of the most important contributors to genetic diversity and often associated with diseases and cancers [1–5]. To reveal the landscape of genetic variation in human or various organisms, several methods including next-generation sequencing and paired-end mapping have been developed to detect and characterise GSV [6]. However, the relationship between GSV and phenotypic consequence remains unclear. Efficient approaches to induce targeted GSV are crucial for studying this relationship and for practicing personalized gene therapy. Approaches to generate targeted translocations [7, 8], deletions [5], or duplications and inversions [5] of genomic segments in human cells have been developed by generating two double strand breaks (DSB) utilizing two pairs of engineered ZFN. But the outcomes of induced GSV in a population varied greatly among the progeny, suggested that the non-homologous end joining (NHEJ) repair of ZFN induced DSB would produce multiple, unpredicted mutations. Therefore, induction of GSV directly using this strategy is undesirable in fundamental studies and risky in gene therapy. Oligonucleotide-based gene modification is a site specific genome editing strategy utilizing triplex-forming oligonucleotide or single-stranded oligodeoxynucleotide and has been shown to successfully mediate modification of genomic DNA in mammalian cells in a precise sequence-specific manner [9], but with much lower efficiency.

Generally, it is challenging to generate custom ZFNs with high specificity and activity from non-commercial sources. TALENs, a recently developed genome editing method, has proved to be a more accessible genetic manipulation tool since TALENs can readily be manufactured in academic research labs with common existing molecular biology reagents and expertise [10]. We previously showed that two pairs of TALENs could also induce a heritable 792 bp chromosomal deletion in silkworm, *Bombyx mori*, in a similar manner of ZFN in human cell lines [11]. Targeted 7 kb deletions and inversions using TALEN were also demonstrated in livestock [12]. These studies suggested that TALEN can serve as a more powerful tool to induce targeted GSV. Also, various strategies to construct custom TALEN have been established, including standard restriction enzyme and ligation-based cloning, ‘Golden Gate’ cloning, solid-phase assembly and ligation independent cloning technique for both single TALEN construction and high throughput assembly [13–15]. Generating an active TALEN pair is feasible for any researcher who is familiar with conventional molecular cloning. We proposed that a combination of TALEN induced DSB and Oligonucleotide-based gene modification induce targeted GSV in a predictable, precise sequence specific manner.

To verify our hypothesis, we constructed an active TALEN targeted to an endogenous gene (*Bm702*), which is 8.9 Mb downstream *BmBlos2* and induced targeted GSV in silkworm embryos. Our results revealed that: (1) simultaneous expression of two TALENs could induce targeted genomic deletions, inversions and duplications, and (2) addition of ssODN could reduce unwanted mutations. In addition, delivery of additional TALEN into the organism may increase the possibility of off-target cleavage, which is the major concern of hybrid nucleases mediated genome editing. We also tested the possibility of chromosomal deletions using one pair of TALEN together with an ssODN.

## Results

### High genome editing activity of TALEN-702 *in vivo*

**Figure 1:**
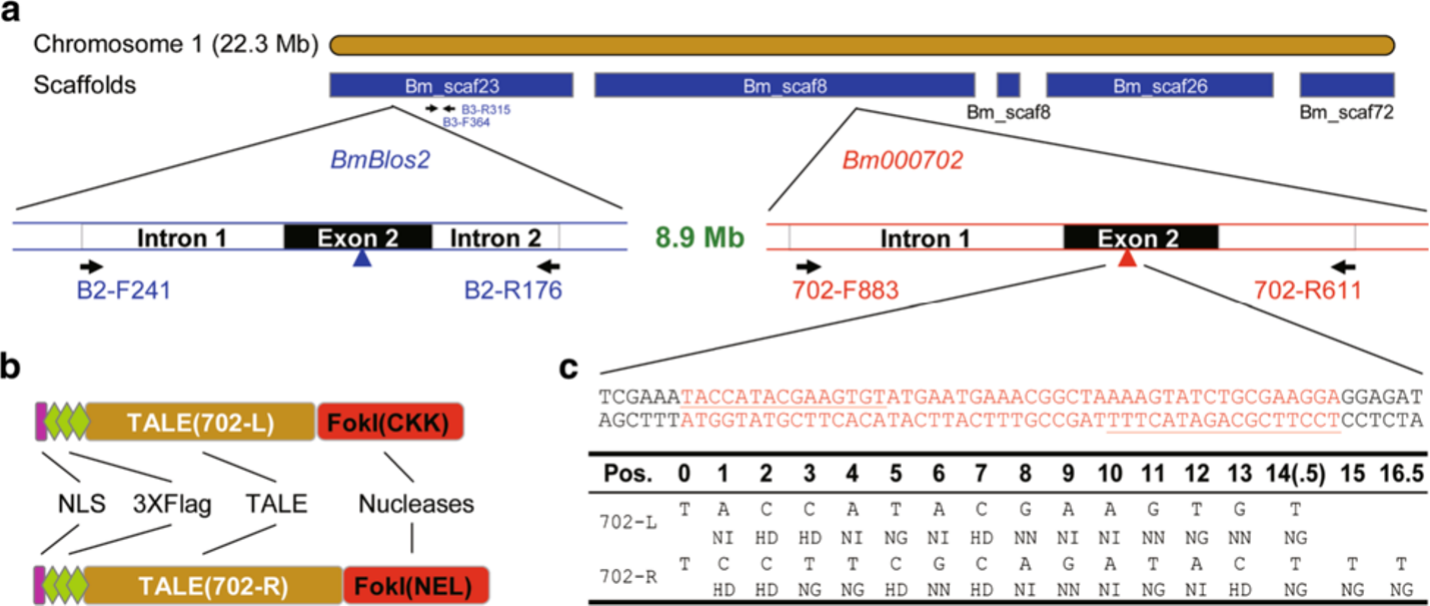
**Design of TALENs that target the BGIBMGA000702 gene. (a)** Schematic representation of the silkworm chromosome 1 and position of BmBlos2 gene and Bm702 gene. Brown box represents the entire chromosome and blue boxes below represent scaffolds mapped to chromosome one. The position of *BmBlos2* gene and *Bm702* gene are indicated by oblique lines. Exon 2 of each gene is indicated by a black box. Blue and red arrows represent the position of TALEN recognition sites for *BmBlos2* gene and *Bm702* gene, respectively. Black arrows and letters below them represent the position and names of primers used to detect mutations. **(b)** Schematic representation of TALEN proteins. A triple flag tag (light green boxes) and a nuclear localization signal (NLS) (purple boxes) are fused to TALE domain (yellow boxes) of each TALEN monomer. The *Fok*I domain (red boxes) is linked to the TALE domain through a flexible linker. **(c)** TALEN target sequences and the corresponding RVDs within each repeat. The entire TALEN recognition sequence is shown on the top, in which underlined sequence represents the recognition site of the corresponding TALEN monomer. RVDs utilized for each TALEN monomer are shown on the bottom. The number at the top of the left panel indicates the position of the repeat. The letters at the top of each TALEN monomer represent the target sequence and the letters below represent the RVDs of the corresponding repeat.

**Figure 2:**
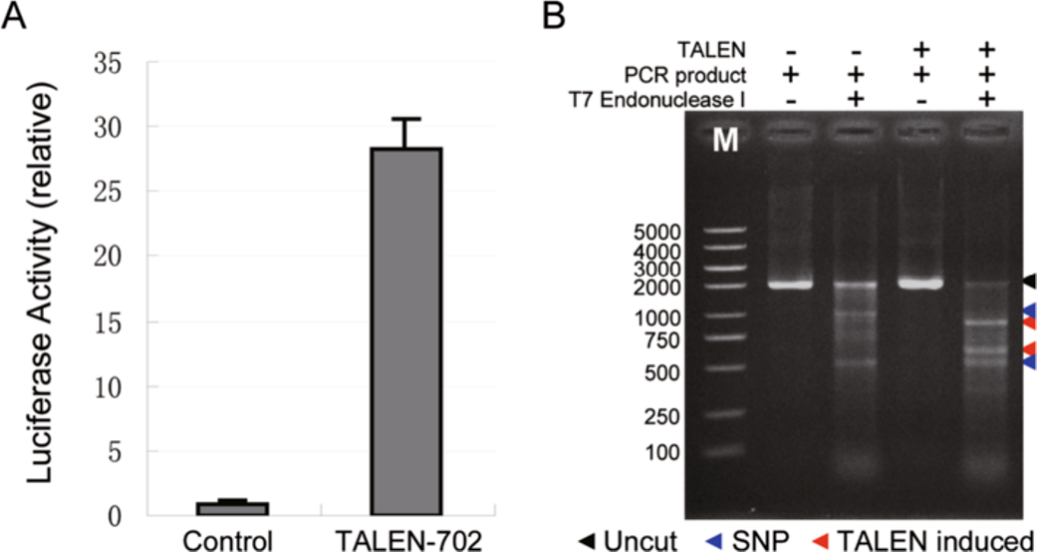
**Genome editing activity of TALEN-702. (A)** *In vivo* analysis of TALEN activity in HEK 293 T cells using a luciferase reporter system. The entire TALEN-702 recognition site was cloned into a pSSA-Luc vector and transfected into HEK 293 T cell with (TALEN-702) or without (control) *in vitro* transcribed TALEN-702 mRNA. Luciferase activity was detected 24 hours post transfection. **(B)** Transient assay of TALEN activity in silkworm embryos using T7EI assay. PCR was performed using primer 702 F-883 and 702R-611 using genomic DNA from un-injected embryos (lane 2 and 3) or injected embryos (lane 4 and 5). M represents DNA ladders, the letters on the left indicate length of each band.

Previously we have constructed two active TALENs targeting the 2^nd^ exon and the 3^rd^ exon of *BmBlos2*, termed TALEN-B2 and TALEN-B3 in this study. The results showed that simultaneous expression in silkworm embryos could generate heritable 792 bp chromosome deletions [11]. To test whether TALEN can induce GSV such as genomic deletions, inversions and duplications with a large chromosomal fragment, we selected *Bm702*, an endogenous gene located 8.9 Mb downstream of *BmBlos2* as a target gene to generate additional TALEN (Figure 1a). To test the activity of TALEN-702, a 48 bp recognition site was cloned into the pSSA-Luc vector. The insertion site was between two truncated luciferase fragments. The vector was transfected into human embryonic kidney (HEK) 293 T cells with or without transcribed TALEN-702 mRNA *in vitro*. Luciferase activity of co-transfection of TALEN-702 and SSA reporter vector detected 24 h post-transfection showed a 28-fold increase, compared with transfection of SSA reporter vector only (Figure 2A), which suggested that TALEN-702 has a high activity to promote homologous recombination (HR) events in HEK 293 T cells. As SSA recombination assay is based on the HR repair pathway, and targeted GSV relies on the NHEJ repair pathway, T7 Endonuclease I digestion (T7EI) assay was then conducted to demonstrate that the repair of TALEN-induced DSB was through the NHEJ repair pathway. TALEN-702 mRNA was microinjected into about 100 silkworm embryos. To detect NHEJ events, PCR product using primers flanking the endogenous TALEN-702 cleavage site (702 F-883 and 702R-611, Additional file 1: Table S2) was subjected to T7 Endonuclease I digestion. The length from forward and reverse primer to TALEN-702 cleavage site is 883 and 611 bp, respectively, so the PCR product can be digested into two fragments (883 and 611 bp) only if NHEJ events occur at TALEN-702 cleavage site. The result showed that TALEN-702 could induce efficient mutations in the endogenous gene loci through NHEJ repair pathway (Figure 2B). However, the accurate mutation frequency can not be measured because of the existence of SNP bands (about 1000 and 500 bp, Figure 2B).

### Large chromosomal structure variation mediated by TALEN-B2 and TALEN-702

**Figure 3:**
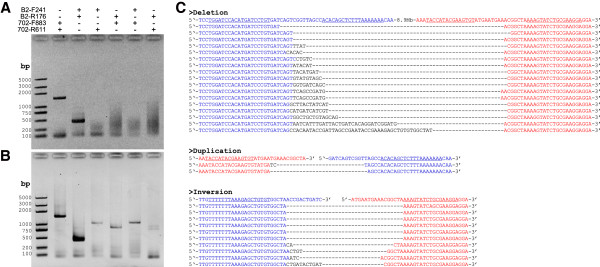
**Detection of large chromosomal structural variation.** **(A and B)** PCR was performed using primer set to detect chromosomal deletion (lane 4), duplication (lane 5), and inversions (lane 6 and 7) from genomic DNA of un-injected embryos **(A)** and injected embryos **(B)**. For comparison, primer sets to amplify the flanking regions of TALEN-702 site (lane 2) and TALEN-B2 site (lane 3) were also used. M represents DNA ladders, the letters on the left indicate length of each band. **(C)** Sequences of the PCR products. The wild-type sequence is shown at the top with the full TALEN-B2 site in blue and the TALEN-702 site in red. The TALEN recognition sites are underlined, and deletions are indicated by dashed lines.

Previously we have shown that co-expression of TALEN-B2 and TALEN-B3 could induce a heritable 792 bp chromosomal deletion [11]. To test whether such deletion can occur between two DSBs whose distance is much larger, and whether co-expression of two distant TALENs can induce chromosomal inversions and duplications, *in vitro* transcribed mRNA of TALEN-B2 and TALEN-702 were mixed and microinjected into about 100 silkworm embryos. PCR was performed using genomic DNA from pooled embryos 3 day after microinjection and specific primers to detect genomic deletions, duplications and inversions. As shown in Figure 3, all primers designed to detect 8.9 Mb deletions, duplications and inversions could amplify products with expected size from genomic DNA of injected embryos (Figure 3B), but not un-injected (Figure 3A). Compared with bands of native locus, bands of induced GSV products are much weaker, but they still can be detected easily at various PCR conditions (data not shown), indicating a considerable frequency. To further confirm the targeted GSV, we cloned the PCR products and determined their sequences. The results showed that 5′ portion of the 2^nd^ exon of *BmBlos2* was directly joined to 3′ portion of the 2^nd^ exon of *Bm702* in the deletion events; 5′ portion of the 2^nd^ exon of *Bm702* was directly joined to 3′ portion of the 2^nd^ exon of *BmBlos2* in duplication events; 5′ portion of the 2^nd^ exon of *BmBlos2* were joined to inverted 5′ portion of the 2^nd^ exon of *Bm702* in inversion events (Figure 3C). In all the events, small indels were observed at the breakpoint junctions, suggesting that TALEN-induced GSV through NHEJ repair pathway is not a precise approach.

### Precise GSV using TALEN and ssODNs

**Figure 4:**
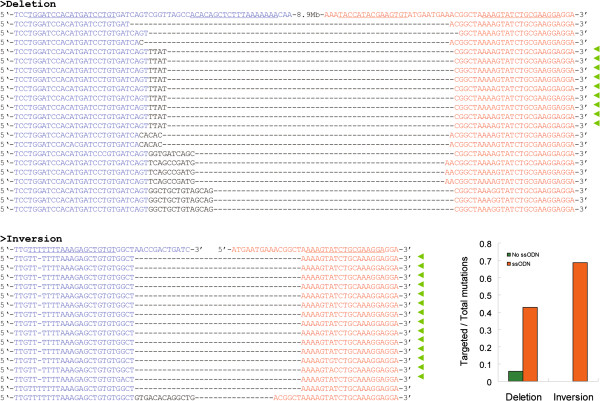
**Precisely chromosomal deletions and inversions using TALEN and ssODNs.** B702-IN and B702-DE were injected with a mixture of two TALENs separately. Sequences are aligned as described in Figure 3C. Sequences equal to designed ssODNs were labelled by green arrows and their percentages among total sequenced clone were shown in the column chart.

In most cases, mutations such as small indels are not wanted. To verify our hypothesis that introduction of a well designed ssODN will guide the targeted GSV into a more precise manner, two ssODNs (B702-DE and B702-IN, Additional file 1: Table S2) were designed and synthesized. To distinguish ssODN guided modifications from natural DNA repair pathways, an additional sequence “TTAT” was introduced into the B702-DE and a nucleotide (T) was deleted in the right TALEN-B2 binding site of B702-IN. B702-DE was co-injected with TALEN-B2 and TALEN-702 into about 100 silkworm embryos. PCR application was performed using specific primers to detect potential deletions from genomic DNA of injected embryos. Similarly, B702-IN was co-injected with TALEN-B2 and TALEN-702 and PCR was performed to detect potential inversions. The PCR products were cloned and sequenced. Compared with injection of TALEN-B2 and TALEN-702, the addition of ssODN significantly reduced the unwanted mutations (Figure 4).

### Heritable chromosomal deletions using a pair of TALENs and ssODN

**Figure 5:**
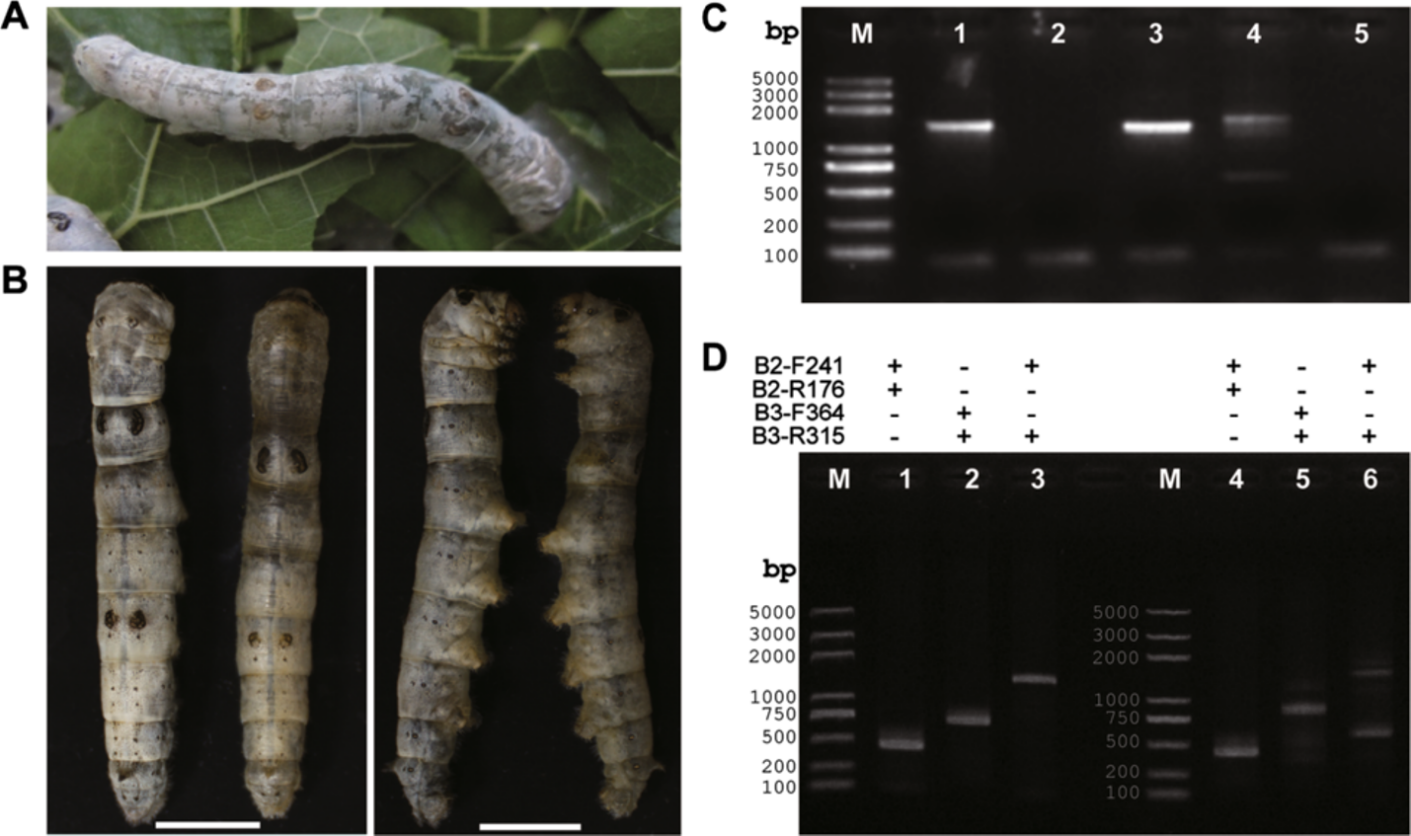
**Large chromosomal deletions using a pair of TALENs and ssODN. (A)** Mosaic mutations induced by co-injection of TALEN-B3 and ssODN. Silkworms were reared on fresh mulberry leaves. **(B)** Images of germline mutations from the dorsal side (left panel) and the lateral side (right panel). The worm on the left in each panel is wild type silkworm. Silkworms were reared on artificial diet. The scale bar represents 1 cm. **(C)** Gel analysis of PCR amplifications using primers B2-F241 and B3-R315 from genomic DNA of 5 G1 mutant individuals (numbers 1 to 5). M represents the DNA ladders, the letters on the left indicate length of each band. **(D)** Gel analysis of PCR amplifications using genomic DNA of wild type (panel indicated as 1 to 3) and G2 mutant silkworm from NO. 4 G1 mutants (panel indicated as 4 to 6). Primer sets are shown on the top of each panel.

Although TALEN was reported to be sequence-specific, off-target cleavages were also observed [16]. We have demonstrated that co-expression of two TALENs could induce targeted GSV, however, this strategy involves the introduction of an additional TALEN as compared to the conventional site directed mutagenesis, which uses only one TALEN. In our case, any combination of the four half target site of TALEN-B2 and TALEN-702 don’t have homologous sequences within the entire silkworm genome, so we didn’t check the off-target cleavage activity. In general, involving of an additional TALEN would of course greatly increase the possibility of off target cleavage, even when obligated *Fok*I variants are utilized. Chen *et al*. [17] used ssODN in tandem with a pair of ZFN to efficiently produce human cell lines with targeted genomic deletion in a defined manner. To perform large precise deletions through TALENs, we tested the possibility of combining TALEN with ssODN. An ssODN composed of a 60 nt sequence distal (794 bp) to the B3 cleavage site, followed by a 40 nt sequence containing B3 right site (Additional file 1: Table S2), was synthesized and co-injected with TALEN-B3 mRNAs into 402 silkworm embryos. Only one mosaic mutation was observed from 35 hatched G0 silkworm larvae (Figure 5A, Additional file 1: Table S1). Twenty one G0 larvae successfully developed to moths to produce 11 G1 silkworm broods and 21 moths were subjected to genomic DNA extraction and PCR amplification of the fragment containing two target sites and intervening sequences. Interestingly, we observed a small amplified DNA segments with the expected size from the mosaic, but not the other 20 silkworms (Additional file 1: Figure S1). Sequencing of the weak band indicated that a 794 bp segment was deleted from the genome as expected.

### Genetic and molecular analysis of ssODN-mediated mutations

To test whether this ssODN mediated 794 bp chromosomal deletions was heritable, about 3000 silkworms from 11 G1 broods were reared and allowed to develop to the 3^rd^ instar. Five silkworms with translucent skin phenotype were obtained from the brood whose male parent was mosaic (Figure 5B). This was in accordance with that mosaic G0 moths are more likely to produce mutant G1 individuals, which we also observed in our previous studies [11]. The results suggested that ssODN mediated chromosomal deletions was heritable, although at a very low frequency (about 9.1% for broods and 1.7% for individuals). To check whether the G1 mutants were caused by TALEN-B3 only or TALEN-B3 together with ssODN, 5 G1 mutants were allowed to develop to moths and subjected to genomic DNA extraction after mating. Genomic PCR (primers: B2-F241 and R315) results (Figure 5C) showed that two individuals (NO.1 and NO.3) were small indels induced by TALEN-B3 only. Neither native bands nor deletion bands were detected in two individuals (NO.2 and NO.5), suggesting that there might be some unknown complex chromosomal rearrangements at this locus in these two individuals. Interestingly but unfortunately, these two individuals failed to produce any offspring, and genomic DNA from G1 individuals were not good enough to perform more experiments such as southern blotting to determine the exact sequence change in this region. To our surprise, two bands were detected in one individual (NO.4), the smaller one of which was with the same size as the deletion band and the larger one was slightly larger than the native band (Figure 5C and D). As all the 5 G1 mutants were female and should have only one Z chromosome, we suspected that NO.4 individual might also be a consequence of a complex chromosomal rearrangement event.

## Discussion

Approximately 5% of the human genome was defined as structurally variant in the normal population, involving more than 800 independent genes [18]. Structural variation can comprise millions of nucleotides of heterogeneity within every genome, and are likely to make an important contribution to human diversity and disease susceptibility [19]. As the rapid progress of genome sequencing and bioinformatics tools, GSV has been identified in more and more genomes of various organisms including animals and plants. Targeted induction of certain GSV such as deletion, duplication and inversion is one of the most important tasks of genome editing for both analytic and therapeutic purposes. Compared with conventional gene targeting such as site directed mutagenesis (knockout), gene correction and addition, targeted GSV can be used to reveal the relationship between natural occurred GSV and its phenotypic impact, and to be used as powerful genetic manipulation tools to reengineer large chromosomal segments such as regulatory elements, non-coding RNAs and transgenes.

In recent years, genetic manipulation was greatly revolutionized as the emergence of ZFN and TALEN, as well as the newly emerged RNA-mediated genome editing tools [20–22]. It is much easier and faster to generate site specific knock-out organisms for both model and non-model systems. At the same time, the ability to induce targeted GSV has also been demonstrated in human cells [5, 9, 23, 24] and in tobacco [25] using two pairs of ZFN, in human cells using a pair of ZFN together with ssODN [17], in silkworm and live stock using two pairs of TALEN [11, 12] and in zebrafish using ZFN and TALEN [26]. However, more evidences are needed to address the questions raised by these pioneered studies and those uncovered issues.

Although ZFN and TALEN contain the same *Fok*I nuclease domain and function in a similar way to induce mutations, it was observed that ZFN and TALEN were associated with different mutation patterns [11, 27]. Successful targeted chromosomal deletions, duplications and inversions using ZFN [5, 9, 23–25] prompt us to investigate the possibility of targeted GSV using TALEN. We have previously shown that simultaneous expression of two TALENs could mediate heritable 792 bp segment deletion in silkworm, *B. mori*[11], which was also supported by similar investigations in livestock [12] and zebrafish [26]. In the present study, we generated an active TALEN targeting to an endogenous gene, *Bm702*, which is about 8.9 Mb downstream *BmBlos2* (Figure 1a). Utilizing these two TALENs, we demonstrated that chromosomal deletions and inversions could be obtained at a considerable frequency, and targeted chromosomal duplications were also detected, which was not demonstrated before using TALEN. As there are too many genes in the 8.9 Mb region, it is nearly impossible for us to detect whether these modifications are heritable. However, the observed mutation efficiency should be high enough to generate heritable mutations according to our experience in silkworm. Our results in the present study, together with those reported previously [11, 12, 26], provided an alternative or even better tool for targeted GSV. To reduce unwanted mutations in the targeted GSV outcomes, which are unexpected in both fundamental studies and therapeutic applications, we combined the TALEN-induced GSV and ssODN mediated gene modifications. Despite the fact that not 100% of the mutant sequences were expected, TALEN-induced GSV was observed to work in a more precise manner after introduction of well designed ssODN.

Although at a very low efficiency, it is encouraging to observe targeted chromosomal deletion using TALEN-B3 and B3-794 in somatic cell of silkworm, suggesting that this strategy can also work with TALEN. Unfortunately, none of the five heritable mutants showed the expected genotype. Instead, two of them were mutants generated by TALEN-B3 only and the other three exhibit abnormal chromosomal rearrangements. We speculate that the difference between precise chromosomal deletion demonstrated in human cells [17] and unpredictable outcomes observed in our studies might due to the following reasons. (1) Compared with 5 or 6 bp spacers of ZFN, TALEN-B3 has a much larger size of spacer (16 bp). Generally, larger spacers are easier to generate heterogeneous overhangs than the well defined 4- or 5-bp overhangs produced by ZFN [28]. It is demonstrated that defined overhangs may facilitate targeted insertions of plasmid DNA at genomic sites [29]. (2) Neither ZFN nor TALEN was demonstrated to work with ssODN to generate precise large chromosomal deletions in organisms other than human cell lines. It is possible but not probable that this mechanism doesn’t work in insect species and perhaps other organisms. However, further investigations are needed to demonstrate the above possibilities. This finding also raises a concern about gene therapy, that hybrid nucleases and ssODN, both of which are thought to be or have been used in gene therapy [30, 31], may induce unwanted chromosomal changes.

## Conclusion

In summary, we demonstrated the ability of 2 pairs of TALEN to induce GSV including deletion, duplication and inversion of a chromosomal fragment as large as 8.9 Mb, which is about one third of the entire chromosome, and developed an efficient strategy to reduce the unwanted mutations during structure variation by adding ssODN. Furthermore, it is the first time to show in this study that co-introduction of one pair of TALEN and ssODN may generate complex structure changes other than the expected chromosomal deletion. Although most of the experiments were conducted in silkworm, *B. mori*, a research model Lepidoptera insect, the DNA repair pathways that contend with DNA damage are highly conserved in all living organisms [32, 33], and the mechanism of TALEN-induced mutation is thought to be identical in different biological systems. Thus it is plausible to believe that the strategies developed in this study can be applied to other organisms, and will help understand the biological roles of GSV and therapeutic applications of TALEN.

## Methods

### Design and construction of TALEN-702

The sequence of the 2^nd^ exon of *Bm702* was subjected to a web-based designer tool (http://zifit.partners.org/ZiFiT/Disclaimer.aspx) to design the target site. To avoid potential off-target cleavage, potential sites were aligned to silkworm genome sequence to remove those sequence with homologous in the genome. A 48 bp target sequence was selected, and the corresponding TALEN sequence (TALEN-702 L and TALEN-702R) were generated using backbone as described previously [11] and repeat variable di-residue (RVD) following the rules that NG, HD, NI, and NN recognize T, C, A, and G, respectively (Figure 1b and c). The assembly of TAL effector domain was completed through a commercial service (ViewSolid Biotech).

### mRNA synthesis

Plasmid vectors TALEN-702 L and TALEN-702R were prepared using a plasmid midi kit (QiaGen), digested with *Xba*I (NEB) and phenol/chloroform purified. *in vitro* transcription was performed using MessageMax™ T7 ARCA-capped Message Transcription Kit (Epicentre Biotechnologies). Cap RNA was then polyadenylated using the Poly (A) Polymerase Tailing Kit (Epicentre Biotechnologies). The resulting mRNA was purified using the MegaClear Kit (Ambion) and quantified using a NanoDrop-2000 (Thermo Scientific). mRNA was dissolved in RNAse-free water (Sigma-Aldrich) to a concentration of 500 ng/μl. TALEN-702 L and TALEN-702R mRNA were mixed at a molar ratio of 1:1 and stored at -80°C.

### Luciferase single-strand annealing (SSA) reporter gene assay

The luciferase SSA assay was performed as described elsewhere [15]. The template plasmid containing TALEN-702 target site was generated by PCR-based mutagenesis using a primer containing 48 bp target site at the 5′ portion. The HEK 293 T cell line, which is widely used as an in vitro model system for transfection experiments, was provided by Dr. Fengfeng Zhuang from View-solid Biotech. The use of HEK 293 T cell line was approved by the institutional Ethics Committees of our university and conducted in accordance with the ethical guidelines of the Declaration of Helsinki.

### Microinjection of silkworm embryos

A diapausing strain, Dazao, which is wildly used as a wild type silkworm, was utilized in this study. The larvae were reared with fresh mulberry leaves at 25°C, 75% RH. Parental embryos (P) were incubated at 15°C and 75% RH to produce nondiapusing eggs (G0), which are suitable for microinjection. The microinjections were performed utilizing TransferMan NK2 micromanipulator and Femto Jet 5247 microinjector (Eppendorf) under an SZX16 microscope (Olympus) as we reported previously [11]. For transient assays, embryos were injected within 5 hours after oviposition and harvested 3 days post injections. For germline assays such as heritable chromosomal deletion using TALEN-B3 and ssODN B3-794, embryos were injected within 2 hours after oviposition and allowed to develop to moths. Mosaic mutations were observed as early as the 3^rd^ instar. The resulting G0 males were first crossed with G0 females and the remaining uncopulated G0 males were crossed with wild-type females. The G1 mutations were checked for the translucent skin phenotype on the 3^rd^ instar, and all the positive mutations were allowed to develop to the moths.

### T7 endonuclease I assay

*In vitro* transcribed TALEN-702 mRNA were microinjected into about 100 silkworm embryos. Genomic DNA was extracted from injected embryos using standard phenol/chloroform method. The genomic region encompassing TALEN-702 target site was amplified using primers 702-F883 and 702-R611 (Table S2). The PCR product was treated 5 units of T7 endonuclease 1 (NEB) for 15 min at 37°C and then precipitated by addition of 2.5 volumes of ethanol. The precipitated DNA was analyzed by agarose gel electrophoresis. Genomic DNA from un-injected embryos was used as control.

### Design and synthesis of ssODN

The ssODN was designed as described by Chen *et al.*[17] and synthesized using a commercial service (Genescript). The ssODN was dissolved in RNAse-free water (Sigma-Aldrich) at 100 mM and mixed with corresponding TALEN mRNA for microinjection.

### Sequencing of the mutations

PCR products were inserted into a T-vector (TransGen) and sequenced using a commercial service (Beijing Genomics Institute). The methods used for PCR and T-cloning were followed standard molecular cloning protocols and the instructions provided by the manufacturers.

## Electronic supplementary material

Additional file 1: Figure S1: Chromosomal deletions using TALEN-B3 and B3-794. M represents the DNA ladders, the letters on the left indicate length of each band. The numbers at the top of each panel represent the silkworm hatched from embryos injected with B3 mRNA and ssODN. The main band is from the native BmBlos2 locus with or without the B3-induced small modifications. The expected bands were about 556 bp if large deletions occurred. The numbers at the bottom represent the relative frequencies of large deletions. **Table S1.** Microinjection of TALEN and ssODN into the embryo of Dazao. **Table S2.** Primers and oligodeoxynucleotides used in this study. (DOC 527 KB)
