# Supplementary material for: Multiplex genomic structure variation mediated by TALEN and ssODN
Source: BMC Genomics. 2014 Jan 18;15(1):41. doi: 10.1186/1471-2164-15-41 (PMC3933007; doi:10.1186/1471-2164-15-41)
Supplement: Supplementary file 1 — Additional file 1: Figure S1: Chromosomal deletions using TALEN-B3 and B3-794. M represents the DNA ladders, the letters on the left indicate length of each band. The numbers at the top of each panel represent the silkworm hatched from embryos injected with B3 mRNA and ssODN. The main band is from the native BmBlos2 locus with or without the B3-induced small modifications. The expected bands were about 556 bp if large deletions occurred. The numbers at the bottom represent the relative frequencies of large deletions. Table S1. Microinjection of TALEN and ssODN into the embryo of Dazao. Table S2. Primers and oligodeoxynucleotides used in this study. (DOC 527 KB) [file 12864_2013_7005_MOESM1_ESM.doc]

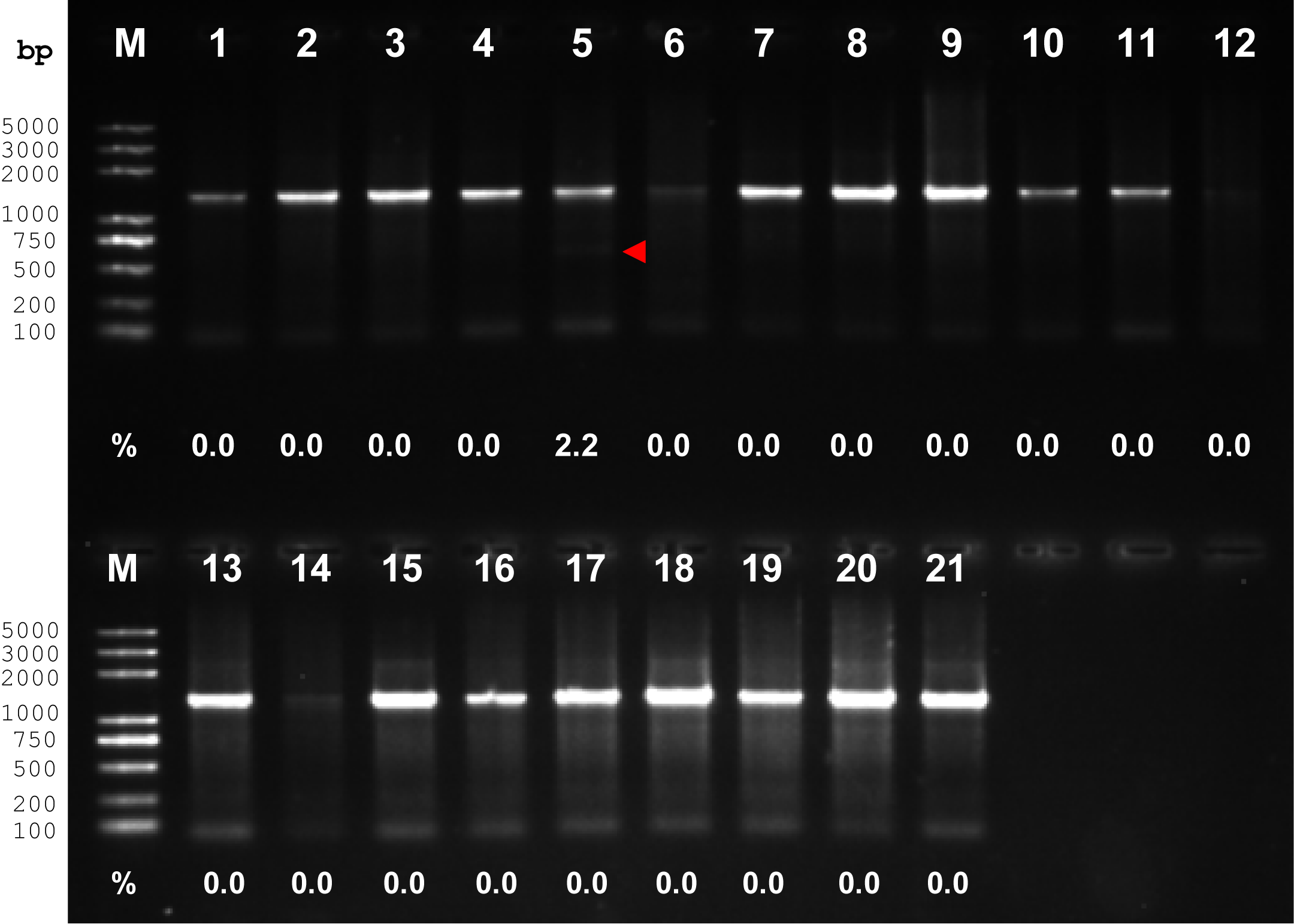


**Supplementary Figure 1** Chromosomal deletions using TALEN-B3 and B3-794. M represents the DNA ladders, the letters on the left indicate length of each band. The numbers at the top of each panel represent the silkworm hatched from embryos injected with B3 mRNA and ssODN. The main band is from the native BmBlos2 locus with or without the B3-induced small modifications. The expected bands were about 556 bp if large deletions occurred. The numbers at the bottom represent the relative frequencies of large deletions.

**Supplementary Table 1** Microinjection of TALEN and ssODN into the embryo of Dazao.

| **strains** | **Injected** | **Hatched** | **5th larvae (mosaic)** | **Mosaic Frequency** | **G1 broods (positive)** | **Positive larvae** |
| --- | --- | --- | --- | --- | --- | --- |
| Dazao | 402 | 61 | 35(1) | 0.03 | 11(1) | 5 |

The percentage shown in the fifth columns were derived using the numbers in the column to the left. The numbers in the “()” were used as numerator and the numbers to the left were used as denominator.

**Supplementary Table 2** Primers and oligodeoxynucleotides used in this study.

| Names | Sequences | Purpose |
| --- | --- | --- |
| >B2-F241 | TTGGTCCAGTAGGTTTGAAGTAGGT | PCR amplification and sequencing |
| >B2-R176 | ATCCTGATTAACCTAGTTTACACACAT | PCR amplification |
| >702-F883 | TTTGATGCTATTTAACTGGGTGC | PCR amplification |
| >702-R611 | GTTCCACTGCGATACTGTGTTCTC | PCR amplification |
| >B3-F364 | TCCAATTTGAGGGCAATGCTAC | PCR amplification and sequencing |
| >B3-R315 | ATTTCACCACCTCATTCAACTAAGAT | PCR amplification |
| >B3-794 | TGTCACCGAGCTGTTCGAGTTTCGAAGTCCTGGATCCACATGATCCTGTGATCAGTCGGTGCCGCGAACCTGAACAAGACCCTTAATGAATACAACGAGA | Large chromosomal deletion |
| >B702-DE | TGTCACCGAGCTGTTCGAGTTTCGAAGTCCTGGATCCACATGATCCTGTGATCAGTTTATCGGCTAAAAGTATCTGCGAAGGAGGAGATCGGGGATATCAA | Precise deletion |
| >B702-IN | GTCCAGCAACCATTTCAGCCTGAAGGTAGTCAGTTGTT-TTTTAAAGAGCTGTGTGGCTAAAAGTATCTGCGAAGGAGGAGATCGGGGATATCAA | Precise inversion |

Sequences were written from 5’ to 3’. TALEN binding sites were underlined
